# Lacunar cerebral infarction following endovascular interventions for phlegmasia cerulea dolens

**DOI:** 10.1016/j.jvscit.2021.06.010

**Published:** 2021-07-01

**Authors:** Ashley M. Otto, Maeghan L. Ciampa, Eric D. Martin

**Affiliations:** a F. Edward Hebert School of Medicine, Uniformed Services University, Bethesda, Md; b Department of Surgery, Dwight D. Eisenhower Army Medical Center, Fort Gordon, Ga

**Keywords:** Phlegmasia cerulea dolens, Endovascular, Lacunar infarct, Thrombectomy, Deep venous thrombosis

## Abstract

Phlegmasia cerulea dolens is a rare presentation of deep venous thrombus treated with catheter directed thrombolysis and pharmacomechanical thrombectomy. This is the case of a 78-year-old woman who underwent catheter directed thrombolysis to treat phlegmasia cerulea dolens and subsequently developed left-sided hemiplegia and expressive aphasia in the setting of an international normalized ratio of 2.0. Further imaging revealed a lacunar infarct in the right thalamus with a middle cerebral artery distribution. Further workup revealed a patent foramen ovale. We highlight the unexpected enigmatic consequence from multimodal endovascular intervention, the consequence of long-term inferior vena cava filters.

Phlegmasia cerulea dolens (PCD) is a limb-threatening presentation of deep venous thrombosis (DVT) associated with high morbidity and mortality due to the ensuing venous hypertension that results in venous gangrene, and is commonly associated with iliofemoral DVTs.^1^ The mainstay of treatment is endovascular interventions: catheter directed thrombolysis (CDT) and pharmacomechanical thrombectomy.^1^, ^2^, ^3^, ^4^ The cause of PCD is multifactorial with inferior vena cava (IVC) filter thrombosis as one proposed risk factor.^3^^,^^5^

The mechanism of lacunar infarcts is classically taught to be a result of microvascular changes of small cerebral arterioles rather than emboli.^6^ Patent foramen ovale (PFO) is present in up to 25% of the population and is the source for up to 50% of emboli.^7^ Here we report a 78-year-old woman with thalamic stroke after multimodal endovascular intervention for PCD. Consent for publication was obtained from the patient.

## Case report

The patient is a 78-year-old woman who presented to emergency department with 2 days of gradually worsening right lower extremity (RLE) swelling, pain, and paresthesia that was limiting mobility. She was independent with a clinical frailty score of 2. Her past medical history was significant for diabetes mellitus with Hgb a1c 10.5 and a provoked left lower extremity DVT 30 years earlier treated with a course of warfarin and permanent IVC filter placement. She denied prior hypercoagulability workup and reported that her first episode of thrombosis followed a prolonged airplane ride. She was not anticoagulated at the time of presentation. She denied a history of dysrhythmias, prior embolic events, recent travel, injury, surgery, illness, or known exposure to COVID-19. She denied a family history of blood clots or hypercoagulability.

Physical examination revealed an RLE that was severely taut with cyanosis with nonpalpable peripheral pulses and multiphasic signaling on Doppler and tenderness to the mid-thigh. The contralateral limb was soft, nontender with color appropriate of ethnicity and palpable peripheral pulses. An RLE venous duplex ultrasound scan was performed with noncompressible veins and loss of spontaneous flow from the right popliteal vein extending to the right common femoral vein consistent with DVT. On presentation, she did not display hemodynamic instability nor complained of cardiopulmonary symptoms.

**Fig 1:**
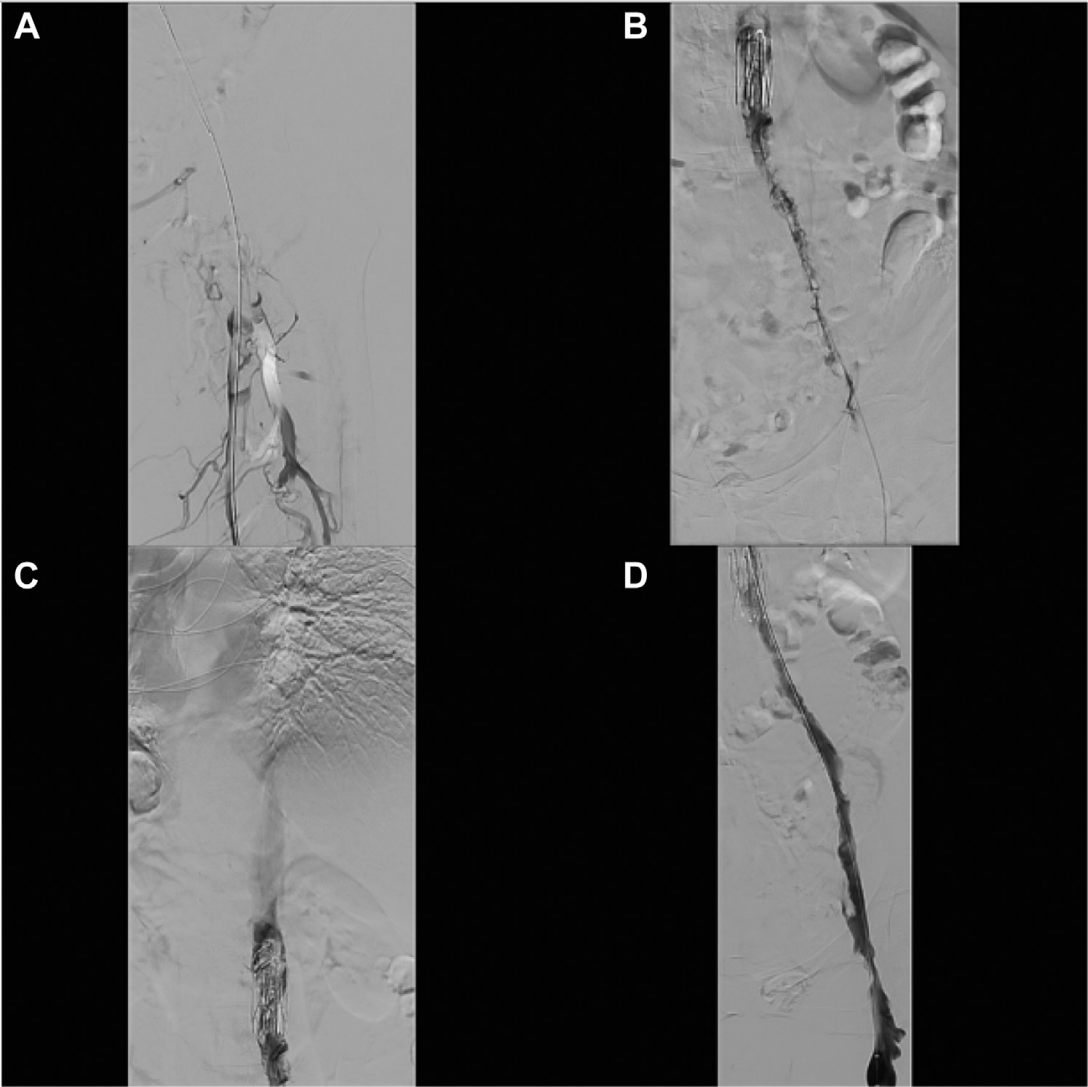
Initial attempt at revascularization. **A,** Significant thrombus in the common femoral vein. **B,** Significant thrombus in the right common iliac and inferior vena cava (IVC) with the IVC filter. **C,** Thrombus in the IVC filter without flow proximal to IVC. **D,** Recanalization of the right common iliac and IVC at completion of the initial procedure. UniFuse catheter in place.

**Fig 2:**
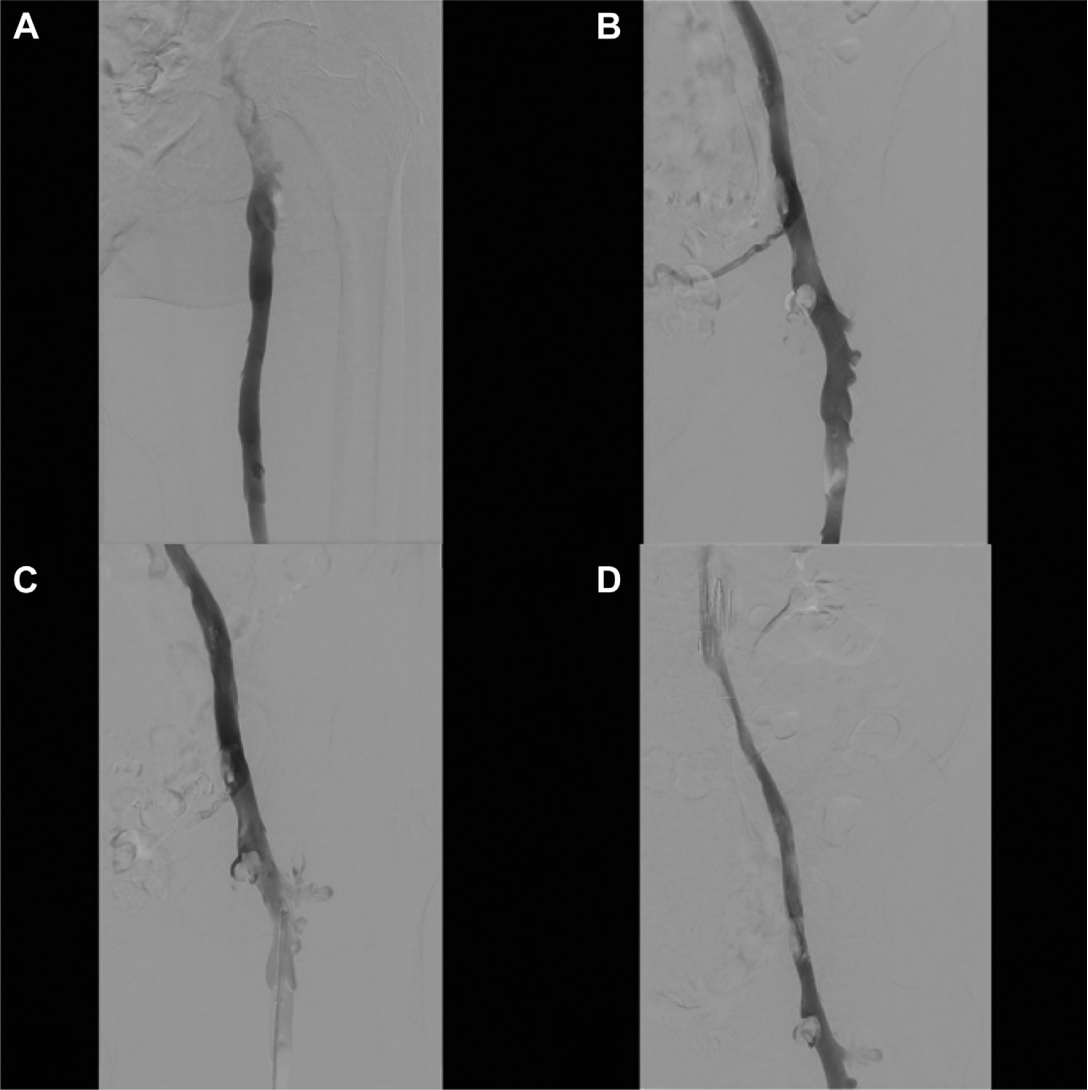
Completion venogram, suction thrombectomy, and balloon venoplasty. **A,** Initial venogram performed on return to the IR suite showed recanalization of the common femoral vein. **B,** Initial venogram of the right common femoral vein revealed a residual clot. **C,** Suction thrombectomy of residual clot burden. **D,** Completion venogram demonstrates recanalization proximal to the inferior vena cava (IVC) filter. *IR*, Interventional radiology.

Because of progressive symptoms concerning for PCD and imminent development of venous gangrene in a patient with good functional status, she was admitted directly to the angiography suite for CDT and pharmacomechanical thrombectomy with tissue plasminogen activator (tPA) and heparin. Angiogram revealed thrombus extending proximal to the IVC filter in the mid-IVC (Fig 1). She was transferred to the intensive care unit for continued CDT with tPA (1 mg/h) and heparin (500 u/h) through a Uni∗Fuse catheter. tPA was discontinued overnight for a fibrinogen of 82 mg/dL. The next morning, she reported subjective improvement of symptoms and return of palpable peripheral pulses with resolution of pain. She returned to the angiography suite for catheter removal and completion venogram that revealed persistent thrombus burden, and the decision to remove the remainder of the clot was made to decrease the risk of recurrence. Using the Penumbra Indigo CAT8 system (Penumbra, Alameda, Calif), mechanical suction thrombectomy, and balloon venoplasty were performed with successful recanalization (Fig 2). She was transferred to the floor, heparin was discontinued, and rivaroxaban initiated.

**Fig 3:**
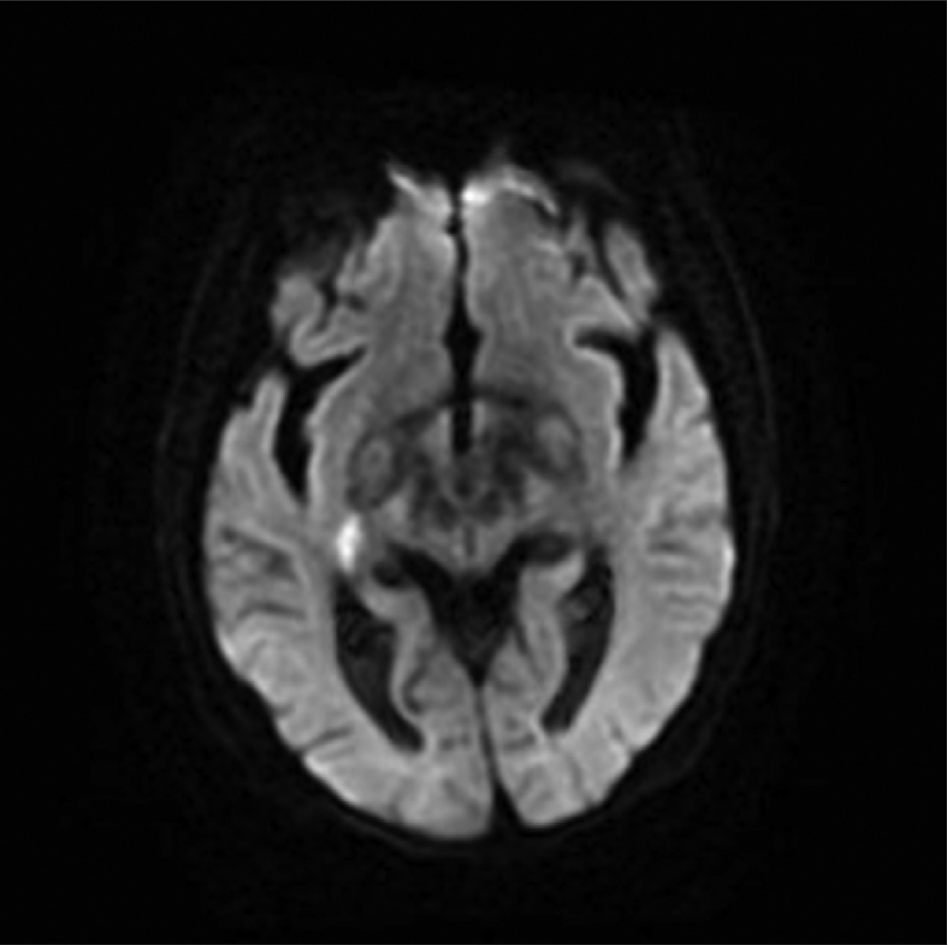
Lacunar infarct: focal area of restricted diffusion within the right thalamus in the area of the internal capsule and basal ganglia.

On postoperative day 2, she was noted to have expressive aphasia and left-sided hemiplegia. A noncontrast computed tomography and computed tomography angiogram of the head and neck were performed without evidence of a hemorrhage or significant carotid or aortic arch atherosclerotic disease. International normalized ratio was therapeutic at 2.0. Given the concern for stroke, a diffusion-weighted magnetic resonance imaging of the brain was obtained (Fig 3) and was notable for the focal area of restricted diffusion within the right thalamus. Because of recent anticoagulation and thrombolysis, she was not a candidate for systemic tPA. Heparin was not reinitiated because of therapeutic international normalized ratio on rivaroxaban. She was transferred to a tertiary center with neurological and neurosurgical capabilities where she underwent a transesophageal echocardiogram with bubble study that revealed a PFO with an atrial septal aneurysm and left-to-right flow noted with color Doppler and a moderate right-to-left shunt during bubble study but no left atrial mass or thrombus. Rivaroxaban was continued and her neurological status improved. She was discharged to a rehabilitation facility with improved left-sided weakness on postoperative day 3. After 2 weeks of rehabilitation, the patient was discharged with a modified Rankin score of 1. Patency of the RLE veins was unknown at the time of discharge, but a repeat duplex ultrasound scan at 8 months after initial presentation showed chronic thrombus in the right common femoral vein, but the RLE venous system remained patent.

Because of multiple episodes of thrombosis, she was placed on lifelong anticoagulation with apixaban. Hypercoagulable disorder was suspected but was deferred by her hematologist as diagnosis would not change plan for management.

## Discussion

PCD is a rare manifestation of DVT that results from complete obstruction of venous return of the lower extremity commonly seen with combined iliofemoral vein occlusion, leading to thrombosis of venous capillaries and eventual ischemia and gangrene.^3^ The presence of PCD is associated with 12% to 50% amputation rate and 20% to 41% mortality rate.^3^ CDT is indicated within 72 hours of symptom onset for patients presenting with PCD secondary to iliofemoral DVT to avoid amputation.^1^^,^^4^^,^^8^ Localized bleeding is the most common complication, accounting for 5% to 11% of patients.^2^ Intracranial hemorrhage after thrombolysis is a catastrophic event that occurs in less than 1% of cases.^2^ Pulmonary embolism (PE) is another commonly feared sequela of CDT. PE has been reported in 30% of acute DVTs.^4^^,^^9^ For patients with DVTs who undergo thrombolysis, the rate of reported PE is 1% to 4.5%, with most discovered incidently.^2^ IVC filter placement decreases incidence of PE in patients with acute DVTs, but increases DVT recurrence and does not improve mortality.^5^^,^^10^ In this patient, the long-term presence of her IVC filter and hypercoagulable state likely contributed to her recurrent DVT and development of PCD.

Paradoxical embolism occurs when a clot within the deep venous system embolizes to the right heart and enters the systemic arterial circulation due to a right-left shunt through a patent atrial septal defect. The prevalence of PFO has been reported in up to 20% to 25% of adults on autopsy and is associated with 40% to 50% of embolic strokes.^7^ The presence of a PFO has been associated with a greater than threefold increase in cerebral emboli after procedures such as pacemaker placement and after DVT.^6^^,^^11^^,^^12^ In this patient, distal embolization to the thalamus likely occurred during or shortly after the second endovascular intervention, resulting in microembolism entering systemic circulation via PFO. PFO closure is recommended in certain patients after stroke.^12^ However, screening for the presence of a PFO as primary prevention has not been studied, and there is no recommendation from the Society of Vascular Surgery or American Venous Forum regarding preoperative screening.

## Conclusions

To preserve limb function in setting of PCD, treatment with endovascular intervention including CDT is performed. Known complications of these endovascular interventions include rare intracranial hemorrhage and PE. However, given the high prevalence of PFO, the risk of paradoxical embolism must be considered a part of the differential in a patient presenting with neurologic symptoms after endovascular venous intervention. The purpose of this case study is to help elucidate the rare enigmatic sequelae of endovascular interventions when treating the deep venous system.
